# Autonomous AI research agents in pancreatic ductal adenocarcinoma immunology: from multimodal evidence to experimental validation

**DOI:** 10.3389/fimmu.2026.1983838

**Published:** 2026-10-09

**Authors:** Yang Li, Yu Li, Rong Huang, Zhi-qiang San, Jun-feng Ye

**Affiliations:** 1 General Surgery Center, First Hospital of Jilin University, Changchun, Jilin, China; 2 School of Nursing, Jilin University, Changchun, China; 3 Department of Ophthalmology, First Hospital of Jilin University, Changchun, Jilin, China

**Keywords:** autonomous AI research agents, multimodal data integration, pancreatic ductal adenocarcinoma, precision oncology, tumor immunology

## Abstract

Pancreatic ductal adenocarcinoma (PDAC) remains one of the most challenging malignancies due to profound biological heterogeneity, complex stromal organization, and limited responsiveness to current immunotherapies. Recent advances in single-cell sequencing, spatial omics, digital pathology, and multimodal profiling have revealed that PDAC immune states are shaped not only by cellular composition but also by spatial organization, treatment context, and patient-specific evolutionary trajectories. However, the increasing complexity and fragmentation of biomedical evidence create substantial challenges for conventional artificial intelligence (AI) approaches that are typically optimized for predefined analytical tasks. Autonomous AI research agents represent an emerging paradigm that extends beyond predictive modeling by integrating scientific reasoning, retrieval of heterogeneous biomedical knowledge, computational tool orchestration, workflow adaptation, and evidence traceability. In this Review, we examine how agentic AI systems may contribute to PDAC immunology research, from characterization of immune ecosystems and spatial cellular interactions to biomarker discovery, neoantigen prioritization, and experimental protocol design. We distinguish AI agents from conventional task-specific models and discuss essential capabilities required for reliable biomedical research, including planning, tool use, verification, memory, provenance tracking, and human oversight. Despite promising technical demonstrations in biomedical domains, direct evidence supporting fully autonomous AI-driven discovery and validation in PDAC remains limited. Therefore, we propose a disease-oriented evaluation framework emphasizing benchmark task design, appropriate human and computational comparators, reproducibility assessment, and experimental validation. Future PDAC-specific AI research agents should prioritize evidence-aware reasoning, multimodal integration, calibrated uncertainty, and transparent computational–experimental workflows. Such systems may ultimately accelerate mechanistic discovery and precision immunology, while maintaining rigorous standards for biological validity, reproducibility, and responsible scientific practice.

## 1 Introduction

Pancreatic ductal adenocarcinoma (PDAC) remains one of the most lethal malignancies worldwide, characterized by aggressive biological behavior, extensive stromal remodeling, profound immune dysfunction, and limited therapeutic responsiveness. Despite substantial advances in cancer immunotherapy, immune-checkpoint blockade has failed to provide broad survival benefits for unselected patients with metastatic PDAC. In the randomized PA.7 trial, the addition of durvalumab and tremelimumab to first-line gemcitabine plus nab-paclitaxel did not significantly improve overall survival or progression-free survival at the population level, highlighting the limitations of applying generalized immunotherapeutic strategies to a biologically heterogeneous disease (1). Recent advances in multimodal profiling technologies have substantially changed our understanding of PDAC immune biology. Large-scale human studies integrating transcriptomics, proteomics, and immune profiling have demonstrated that PDAC tumors contain highly heterogeneous immune landscapes composed of distinct myeloid, stromal, and adaptive immune states (2, 3). Importantly, this heterogeneity extends beyond differences in cellular abundance. Single-cell and spatial transcriptomic analyses have revealed that immune cell states acquire biological significance through their spatial organization, cellular neighborhoods, and interactions with surrounding stromal compartments (4). Multi-region analyses further demonstrate that immune regulatory programs may vary considerably within individual tumors (5), while metastatic lesions can exhibit distinct microenvironmental configurations and lineage-state plasticity (6). These findings indicate that PDAC immune resistance cannot be fully explained by single biomarkers or tumor-wide molecular classifications; instead, it requires integration of cellular states, spatial context, temporal evolution, and treatment history. However, the increasing complexity of PDAC immunological data has created substantial challenges for conventional analytical strategies. Bulk molecular profiling provides valuable population-level information but inevitably averages signals derived from multiple cellular populations. Single-cell technologies resolve cellular states but often lose tissue architecture, whereas spatial approaches restore localization but introduce additional computational and interpretative complexity. Digital pathology, multi-omics datasets, clinical information, and experimental models further contribute complementary but heterogeneous sources of biological evidence. Therefore, the central challenge is no longer simply generating predictive models, but establishing reliable frameworks capable of integrating diverse evidence streams, identifying biologically meaningful patterns, and transforming computational observations into experimentally testable hypotheses.

Artificial intelligence (AI) has already contributed significantly to biomedical research by enabling image analysis, molecular classification, biomarker identification, and large-scale data interpretation. In PDAC, task-specific AI models have demonstrated the ability to uncover clinically relevant heterogeneity. For example, PACpAInt, a deep-learning pathology model trained on large patient cohorts, inferred molecular subtypes from histological images and revealed intratumoral heterogeneity that could be overlooked by conventional bulk RNA-based classification (7). Nevertheless, such models generally operate within predefined objectives, fixed computational workflows, and constrained output spaces. Their value lies in performing specialized analytical tasks rather than independently coordinating the broader scientific process. The emergence of autonomous AI research agents represents a conceptual shift from task-specific prediction toward AI-assisted scientific reasoning and workflow orchestration. Unlike conventional AI models, research agents are designed to interpret objectives, decompose complex questions, retrieve relevant evidence, select appropriate computational tools, execute analytical workflows, verify intermediate conclusions, and maintain traceable records of decision processes. Recent systems such as Biomni, which integrates retrieval-augmented planning with biomedical databases, computational tools, and code execution, and CellAtria, which links data discovery with standardized single-cell analysis workflows, illustrate the potential of agentic AI for coordinating complex biomedical research procedures (8, 9). GeneAgent further demonstrates the importance of external verification by grounding biological interpretations against specialized domain databases (10). Nevertheless, the application of autonomous AI research agents to PDAC immunology remains at an early stage. Current evidence primarily supports technical capabilities, including data retrieval, workflow coordination, and computational assistance, whereas direct demonstrations of AI agents independently generating and experimentally validating novel PDAC immune mechanisms remain limited. Therefore, the critical question is not whether AI agents can replace researchers, but how they can enhance scientific workflows by connecting heterogeneous biological evidence, computational analysis, and experimental validation in a transparent and reproducible manner.

In this Review, we examine the emerging role of autonomous AI research agents in PDAC immunology. We first summarize the biological complexity and multimodal data infrastructure underlying PDAC immune research. We then discuss the architecture and essential capabilities of biomedical AI agents, including planning, retrieval, tool utilization, verification, memory, and human oversight. Finally, we evaluate potential applications across immune microenvironment characterization, spatial immune analysis, biomarker discovery, neoantigen prioritization, and experimental protocol design, while emphasizing current limitations, validation requirements, and future directions toward evidence-aware and reproducible AI-driven biomedical discovery. The conceptual progression from PDAC immune heterogeneity to evidence-aware agentic orchestration and experimental validation is summarized in **Figure 1**.

**Figure 1:**
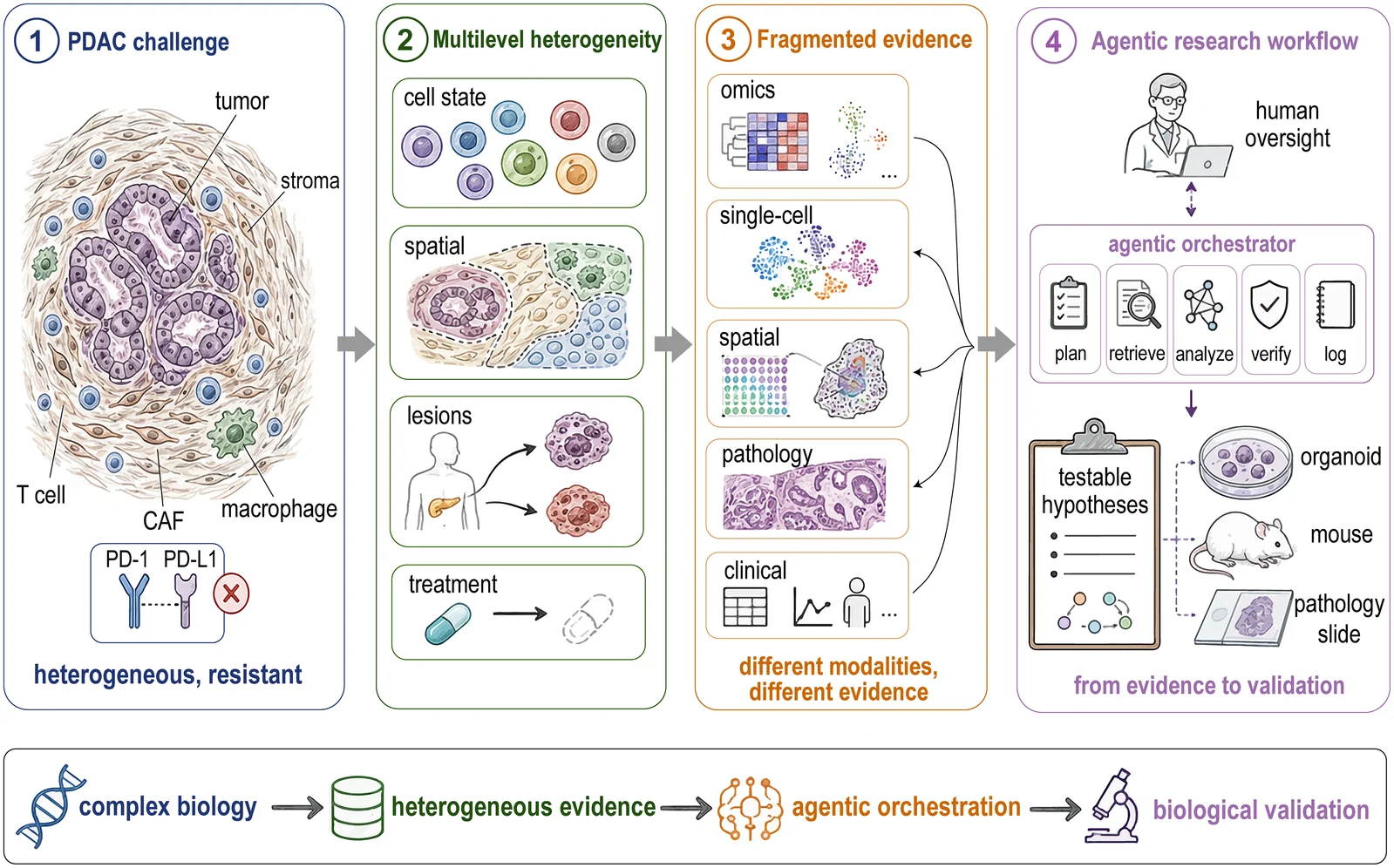
Conceptual framework linking PDAC immune complexity, multimodal evidence, agentic research orchestration, and experimental validation. PDAC immune heterogeneity encompasses malignant, stromal, and immune-cell states, spatial organization, treatment context, and disease evolution. These features are captured through complementary evidence sources, including bulk and single-cell omics, spatial profiling, pathology, clinical data, and experimental models. Evidence-aware AI research agents may coordinate retrieval, analysis, verification, and provenance tracking under human oversight, generating testable hypotheses for subsequent validation in organoid, animal, pathology, and immune-function systems. CAF, cancer-associated fibroblast; PD-1, programmed cell death protein 1; PD-L1, programmed death-ligand 1.

This article is a narrative and conceptual review of autonomous AI research agents in PDAC immunology. Evidence was selected to support the biological problem, define agent capabilities, and examine how agent-generated outputs could be evaluated. We prioritized primary studies of human PDAC, original reports of biomedical research agents and benchmarks, methodological guidance, and official data or knowledge resources. Evidence from non-PDAC systems was included when it established a transferable capability, comparator, or governance principle, and is identified as adjacent rather than disease-specific evidence. Preprints were retained selectively for rapidly evolving agent systems or benchmarks and were explicitly labelled as such. The synthesis distinguishes technical evaluation on pancreatic-cancer data from biological discovery, experimental validation, and clinical utility, which require separate evidence.

## 2 PDAC tumor immunology and data infrastructure

### 2.1 Immune and stromal ecosystem heterogeneity

The label cancer-associated fibroblast conceals biologically important differences in PDAC stroma. Cross-species single-cell analysis identified multiple fibroblast states, including myofibroblastic, inflammatory and antigen-presenting programs, rather than one functionally uniform population (11). These states are not fixed universal lineages, and their distribution varies with local context. Mechanistic perturbation showed that local IL-1-driven LIF-JAK-STAT signaling and TGF-β signaling exert opposing influences on inflammatory and myofibroblastic programs, supporting a dynamic model of fibroblast identity (12). Functional divergence extends beyond transcriptional annotation: experimentally defined pancreatic fibroblast populations can have different effects on tumor growth and antitumor immunity, including a fibroblast lineage associated with immune-supportive activity (13). PDAC stroma is therefore better understood as a set of context-dependent fibroblast states with distinct regulatory and functional properties than as a single uniformly immunosuppressive compartment.

Myeloid measurements require a similar shift from broad labels to state and function. Human PDAC contains macrophage and other myeloid populations whose abundance, phenotype and spatial distribution vary substantially among tumors (14). Multimodal immune mapping likewise resolves patient-specific myeloid configurations that are poorly represented by a simple M1/M2 dichotomy (2). Experimental work on conventional dendritic cells provides a more direct functional anchor: deficient cDC surveillance can impair immune detection and constitute a mechanistic bottleneck in PDAC models (15). Subsequent preclinical intervention further supports the capacity of type 1 conventional dendritic-cell activation to alter checkpoint responsiveness (16). These findings distinguish the abundance of a myeloid population from its competence to support antigen presentation and antitumor immunity.

Adaptive immunity in PDAC is similarly structured by more than a single axis of infiltration. Human single-cell and spatial studies identify prominent T-cell dysfunction and exclusion, but these patterns coexist with tumors containing organized adaptive immune niches (2, 17). Mature tertiary lymphoid structures provide a salient example: in selected PDAC specimens, they form localized sites associated with tumor-specific immune activity rather than a diffuse background of lymphocytes (18). Their presence reinforces the distinction between a population-level pattern of weak checkpoint responsiveness and the immune organization of individual tumors. An adaptive-enriched niche, an excluded T-cell population and a dysfunctional lymphoid state represent biologically different configurations and may therefore generate different mechanistic questions.

Region, treatment context and disease site further reshape the meaning of immune-cell annotations. Multi-region pathology shows that immune-regulatory patterns and myeloid neighborhoods can differ within the same PDAC (5, 14). Single-nucleus and spatial transcriptomic profiling also identified coordinated multicellular states associated with neoadjuvant treatment, while analyses of untreated and treatment-refractory tumors mapped distinct spatial tumor and stromal programs (19, 20). Heterogeneity expands again in metastatic disease. Rapid-autopsy spatial analysis found that primary and metastatic lesions, and different metastatic sites within an individual, may contain different lineage states and microenvironmental configurations (6). Together, these studies make cell state, spatial location, treatment exposure and sampling site part of the biological evidence itself. Any integrative analysis of PDAC immunity must therefore preserve these attributes rather than collapse them into a single tumor-level label. Therefore, PDAC immune heterogeneity represents not only a biological challenge but also an information-integration challenge, requiring AI systems capable of linking cellular states, spatial context, and clinical variables rather than analyzing isolated datasets.

### 2.2 Multimodal evidence landscape and infrastructure requirements for AI agents

No single data modality resolves all of this structure. Bulk transcriptomic and proteogenomic cohorts provide molecular measurements at useful cohort scale but aggregate signals from multiple cell populations and do not retain tissue architecture. Their strength lies in identifying recurrent molecular patterns across cohorts and providing a common frame for comparisons that finer assays may not support at the same scale. Single-cell and single-nucleus assays separate cellular states and support comparisons across populations, yet tissue dissociation or nuclear sampling removes much of the original spatial arrangement (4, 19). Spatial transcriptomics can relocate expression programs within tissue regions, and multiplex imaging can map protein-defined cells and neighborhoods; these approaches identify regional organization and nominate candidate interactions, while functional causality still requires downstream testing (4, 21). Combining transcriptomic, protein-level and spatial measurements can sharpen candidate mechanisms by asking whether a state recurs across measurement layers and whether its proposed neighborhood is anatomically plausible (2, 17). The resulting evidence structure is complementary: bulk data define cohort patterns, single-cell data resolve cellular states, spatial data restore organization, and multimodal agreement strengthens the basis for selecting mechanisms to test.

Functional models determine which candidates can move beyond association, but their value depends on the component of biology retained. Autologous PDAC organoid-immune co-culture can isolate selected tumor-immune relationships and test controlled perturbations, including effects involving suppressive myeloid populations (22). More complex organoid platforms can incorporate T-cell-engaging conditions for immunotherapy testing (23). Conventional patient-derived xenografts preserve aspects of human tumor biology and can support testing with autologous tumor-infiltrating lymphocytes, whereas human stromal and immune contexts are only partly retained (24). Immune-reconstituted orthotopic models add selected human immune components and establish the feasibility of studying pancreatic tumors in a humanized setting (25). These platforms answer different experimental questions rather than forming a simple hierarchy of model sophistication. Organoid co-culture favors controlled perturbation, PDX-based systems retain aspects of patient tumor biology, and humanized models add immune components that can support complementary *in vivo* testing.

Research infrastructure requires the same functional separation. Repositories such as GDC, PDC, GEO and ICGC ARGO provide access to genomic, proteomic or expression datasets, whereas IEDB functions as an immunological knowledge and prediction resource and ClinicalTrials.gov as a clinical-study registry (26–31). These resources differ in interface, authentication, access tier, versioning and evidence type. Analytical tools occupy another layer: CIBERSORTx is a bounded digital-cytometry method, while TIDE remains an influential computational framework although its original web server has been retired (32–34). For reproducible agentic work, resource identity, access status and version must travel with the retrieved evidence. Distributed infrastructure therefore makes orchestration operational: an agent must know what a resource contains, how it can be accessed, what type of output it returns and which provenance accompanies that output. The complementary roles of multimodal data, functional models, and research infrastructure for agent-assisted PDAC immunology are summarized in **Figure 2** and **Table 1**.

**Figure 2:**
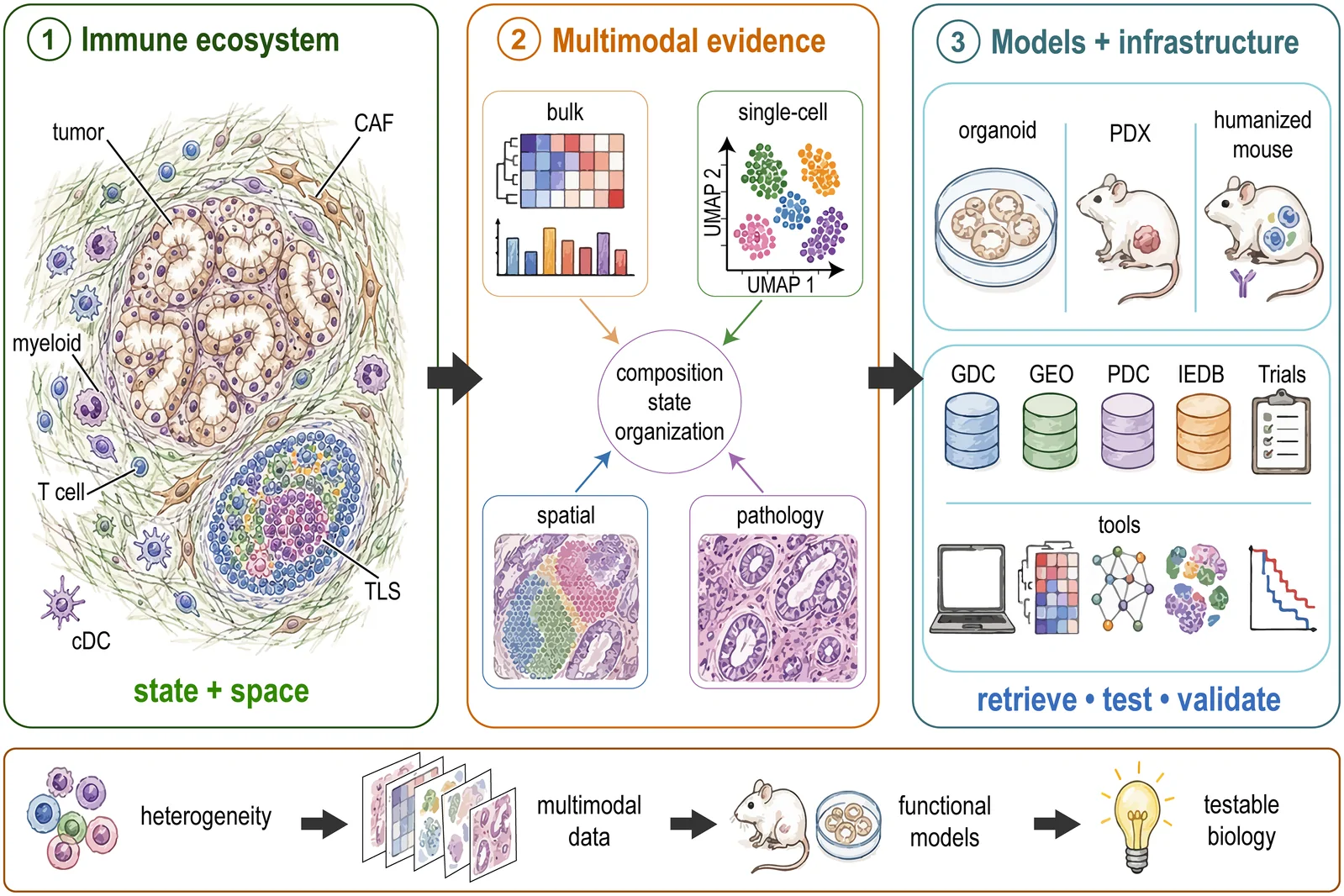
Multimodal evidence landscape and experimental infrastructure for PDAC immunology research. PDAC immune ecosystems comprise heterogeneous malignant, stromal, myeloid, dendritic, and lymphoid cell states organized within distinct spatial contexts. Bulk profiling, single-cell analysis, spatial technologies, and pathology provide complementary levels of biological information, while organoids, patient-derived xenografts, and humanized models support functional validation. Public genomic, proteomic, immunological, and clinical resources provide additional infrastructure for evidence retrieval and analysis. Together, these components connect biological heterogeneity with multimodal integration and experimentally testable hypotheses. CAF, cancer-associated fibroblast; cDC, conventional dendritic cell; TLS, tertiary lymphoid structure; PDX, patient-derived xenograft.

**Table 1:** Data resources for PDAC tumor immunology research.

Data type	Database/resource	Immunology research use	Key considerations
Multi-omics	TCGA-PAAD, ICGC-PACA-CA, CPTAC	Immune infiltration estimation; immune subtype classification	Pay attention to sample size and batch effects
Single-cell/spatial	GEO, Human Cell Atlas, 10x Visium, CODEX/MIBI	Immune cell states; spatial interactions	Harmonize annotation and batch correction
Immune phenotype	TIMER2.0, CIBERSORTx, TISIDB, TIDE	Immune infiltration; immune escape assessment	Algorithm assumptions should be explicitly stated
Antigen/neoantigen	IEDB, TESLA, Cancer Immunome Atlas	Neoantigen prediction; immunogenicity assessment	Validation data are limited; interpret cautiously
Drug/clinical	DepMap, GDSC, ClinicalTrials.gov	Immunotherapy combinations; clinical trial matching	Distinguish approved from exploratory evidence

### 2.3 From task-specific AI to research agents

The transition to research agents should not be framed as a move from simple to powerful AI. Within predefined questions, inputs and outputs, bounded systems already perform demanding PDAC analyses. PACpAInt used histology to infer molecular states across 598 patients and 1,796. Among 77 tumors classified as classical by RNA, 30 contained basal-like regions on other slides, illustrating how a task-specific model can expose spatial heterogeneity obscured by a single bulk label (7). It nevertheless operates within a fixed analytical design: the input, computational path and output space are specified in advance. Agentic systems introduce orchestration across research actions. Biomni combines retrieval-augmented planning with heterogeneous biomedical tools, databases and code execution, whereas CellAtria coordinates data discovery, retrieval and standardized single-cell processing with explicit provenance and logs; the latter has been tested on pancreatic-cancer datasets (8, 9). The distinction lies in how research actions are selected and connected, not in the number of computational steps. A fixed pipeline can be technically elaborate, while an agent may operate within a narrow and carefully guarded task space. For PDAC immunology, the unresolved transition is from performing predefined analyses to assembling provenance-aware evidence, checking intermediate interpretations and directing selected hypotheses toward appropriate experimental tests. Agents therefore extend rather than displace specialized models: the key change is from task execution to research orchestration.

## 3 Autonomous AI research agents: architecture and biomedical evidence

### 3.1 Definition and principles of evidence-aware AI research agents

For this review, we use an operational distinction based on how a system selects and revises research actions. A conventional pipeline follows a prespecified computational path: researchers encode the objective, tool sequence, parameters, and output structure before execution. An AI assistant can retrieve information, write code, or interpret an output, but its next action is usually directed by the user. A research agent can decompose a higher-level objective, select among permitted resources, execute analyses, and alter the next step in response to intermediate results while remaining within a defined objective and permission boundary. The operational discriminator is therefore adaptive research-action orchestration. Goal interpretation, planning, tool selection, state or memory, verification, and human control describe different parts of that orchestration and should be reported separately. The distinction becomes observable when an intermediate result changes which dataset is queried, which analysis is run, or which verification step is required. A long workflow can remain a fixed pipeline, whereas a narrowly scoped agent may adapt meaningfully within a guarded task. This definition separates scientific agency from interface fluency and makes the claimed autonomy open to evaluation.

Verification becomes scientifically useful when it changes the claim or the next action. Internal checks can test code, parameters, or logical consistency; external checks compare an interpretation with a traceable database, analytical method, or study. GeneAgent provides a precedent for domain-grounded verification of gene-set interpretations (10). Traceability alone, however, does not make the evidence independent. A primary report, registry record, and review may represent the same study, while nominally separate analyses may share patients, samples, public datasets, annotations, preprocessing, or model assumptions. An evidence registry should therefore record both provenance and dependency. Concordant findings with a shared lineage strengthen traceability but provide less information than concordant evidence with different key biases (35). When comparable evidence disagrees, the conflict should remain visible. The agent can narrow or downgrade the claim, retrieve evidence with a different dependency structure, request an orthogonal analysis or experiment, or route the issue to human adjudication. This is preferable to manufacturing consensus by counting sources, especially because biomedical contradictions can remain difficult for language models to recognize and preserve (36).

Autonomy is likewise best assigned to actions rather than to an entire system. A system may autonomously select an analysis but require approval before changing a cohort, calling an external service, prioritizing a biological target, or proposing an experiment. The Virtual Lab illustrates computational biological design followed by experiments performed by human researchers (37); Robin couples hypothesis generation and experimental planning to analysis of returned data but retains human review and wet-lab execution (38). These workflows should not be described as if the agent performed the experiments. Reporting who defined the goal, approved tools and data, selected candidates, executed experiments, and interpreted results makes human involvement measurable. The report should also state the timing and consequence of intervention: routine approval, correction of an error, redirection after new evidence, and emergency termination are different forms of control. This action-level account identifies the actual permission boundary. It also distinguishes a human-only comparator from a prespecified human checkpoint within an agent workflow.

### 3.2 Functional architecture for PDAC immunology

A PDAC-specific research agent should organize evidence around the scientific objective and the level of inference it can support. The objective specifies the disease context, cohort or model, permissible actions, and required output. A planner then decomposes the question into retrieval and analytical steps. Tool and data selection is mediated by a resource-and-evidence registry that records modality, patient or model context, access conditions, software or database version, provenance, validation status, and known dependencies. The registry should distinguish resources that contain observations, resources that transform data, and resources that summarize earlier evidence, because these roles create different dependencies. Retrieval and analysis return evidence-labelled outputs such as a patient-level association, a cell state, a spatial neighborhood, or an experimentally supported mechanism. Verification tests whether the output is internally coherent and traceable, whether nominally corroborating evidence is genuinely independent, and whether relevant findings are concordant or discordant. It can accept the claim, qualify its scope, downgrade confidence, initiate further retrieval, request an orthogonal analysis, or seek human review. The resulting record links every conclusion to the data, transformation, and verification that produced it. Biomni demonstrates broad biomedical planning and tool orchestration (8); CellAtria demonstrates provenance-rich discovery, retrieval, and single-cell workflow coordination on cancer datasets that included pancreatic cancer (9); and GeneAgent supplies a verification precedent (10). Together, these capabilities support a proposed architecture rather than a validated PDAC discovery system.

Constrained autonomy makes this architecture useful without obscuring scientific control. Permitted datasets, tools, output types, and external actions should be explicit. High-consequence decisions, including changes to cohort eligibility, acceptance of an inferred mechanism, release of sensitive data, or progression to an experiment, can require prespecified human approval. Parameters with strong biological consequences, such as single-cell quality thresholds, annotation references, spatial-neighborhood definitions, HLA assumptions, and stopping rules, should be guarded or reviewable. The execution record should preserve dataset identities and checksums, access dates where relevant, model and tool versions, prompts or plans, code, parameters, retries, intermediate outputs, evidence dependencies, unresolved conflicts, and human interventions. Such logging allows an analysis to be reconstructed and shows where an adaptive decision changed the workflow. A structured handoff should state the supported claim, unresolved alternatives, requested validation, required controls, and decisions reserved for investigators. It thereby connects computational prioritization to researcher-led experimental design and execution without blurring responsibility. **Figure 3** summarizes this constrained, evidence-aware architecture.

**Figure 3:**
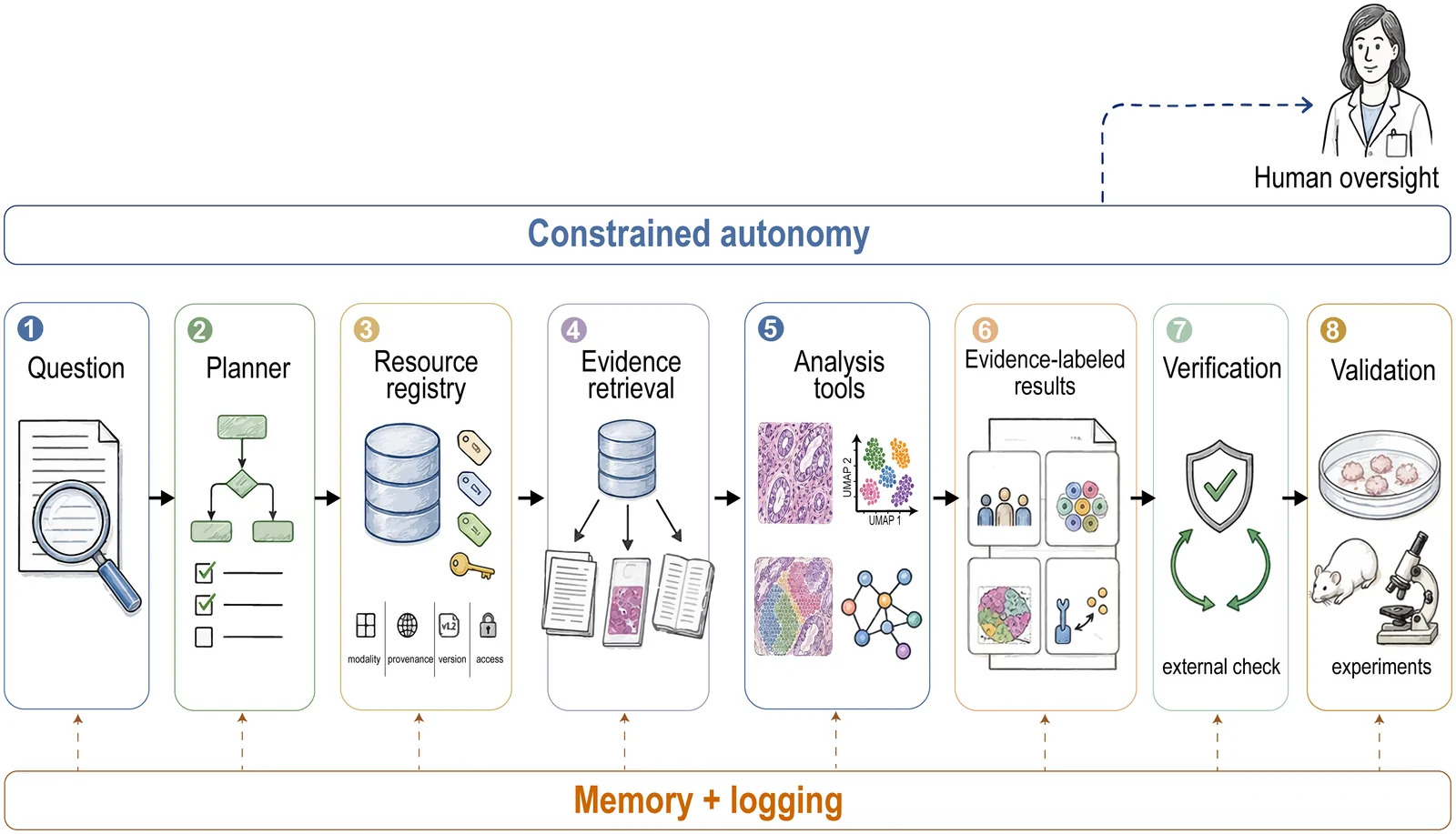
Functional architecture of a constrained, evidence-aware AI research agent for PDAC immunology. A PDAC immunology research question is decomposed into retrieval and analytical tasks through agentic planning. Relevant datasets, literature, and computational tools are selected through a resource registry that preserves provenance, version, modality, access information, and known evidence dependencies. Analytical outputs remain evidence-labeled. Verification assesses provenance, dependency, and discordance and may accept, qualify, or downgrade a claim or route it to further retrieval, orthogonal analysis, or human review before experimental testing. Memory and logging retain sources, parameters, tool calls, intermediate outputs, decision histories, and human interventions throughout the workflow. Prespecified human checkpoints govern high-consequence analytical and experimental actions, defining constrained rather than unrestricted autonomy.

### 3.3 Representative systems and the current autonomy boundary

Representative systems demonstrate complementary capabilities rather than a single ladder of autonomy. GeneAgent embeds domain-grounded checks in biological interpretation (10). Biomni combines retrieval-augmented planning with biomedical databases, tools, and code execution, providing adjacent evidence for broad orchestration but not PDAC-specific validation (8). BioMedAgent similarly supports tool-aware biomedical data analysis (39). CellAtria provides a direct workflow-level connection to the disease area: its evaluation included pancreatic-cancer single-cell datasets and tested data discovery, retrieval, standardization, analysis, and provenance capture. This is direct technical evidence on pancreatic-cancer data, not evidence that the system discovered or experimentally validated a new PDAC immune mechanism (9). SP-Mind provides direct technical task evidence that includes PDAC imaging mass-cytometry data, while its reported task performance does not establish biological or clinical validity (40). For each system, the relevant unit of evidence is the evaluated task and output, not the breadth of the system description.

Other systems clarify the boundary between computational orchestration and experimental research. Co-Scientist coordinates hypothesis generation, critique, ranking, and refinement, with selected hypotheses handed to experimental collaborators; its component analyses also inform evaluation design (41). The Virtual Lab connects multi-agent biological design to human wet-lab validation (37). Robin forms a semi-autonomous loop by analyzing data returned from experiments that humans conduct (38). CRISPR-GPT operates within a bounded gene-editing planning space (42). These systems support distinct claims about verification, planning, iterative reasoning, or experimental handoff. They do not collectively establish a fully autonomous PDAC immunology platform, and the breadth of a system should not be confused with the depth of validation for any one scientific claim. Taken together, these studies support a narrower conclusion: direct technical evaluations on pancreatic or PDAC data have begun, while the strongest transferable evidence for adaptive planning, verification, and returned-data iteration still comes from adjacent biomedical settings. A PDAC research agent can therefore be designed from demonstrated components, provided that each application retains its own evidentiary level, validation route, and human permission boundary.

## 4 Autonomous AI research agents in PDAC tumor immunology: current evidence, transferable capabilities, and validation pathways

PDAC provides a stringent setting for research orchestration because cell states, spatial regions, treatment histories, and metastatic sites carry non-equivalent biological information. A state measured in dissociated cells, a neighborhood observed in tissue, and a response measured after treatment answer different questions even when they concern the same immune population. The applications below are organized across five domains: immune-ecosystem integration, spatial immune ecology, biomarker and treatment-response research, neoantigen prioritization, and experimental design. Four evidence categories are kept distinct throughout: direct technical agent evidence on pancreatic or PDAC data; established PDAC-domain evidence; adjacent biomedical-agent evidence; and prospective applications proposed here. A technical result is described as direct only for the data and task actually evaluated. It does not establish biological discovery, experimental validity, or clinical utility unless those outcomes were tested. This distinction allows each application to be linked to a validation route matched to the biological inference without allowing disease relevance to substitute for agent performance. The five application domains and their corresponding validation pathways are summarized in **Figure 4**.

**Figure 4:**
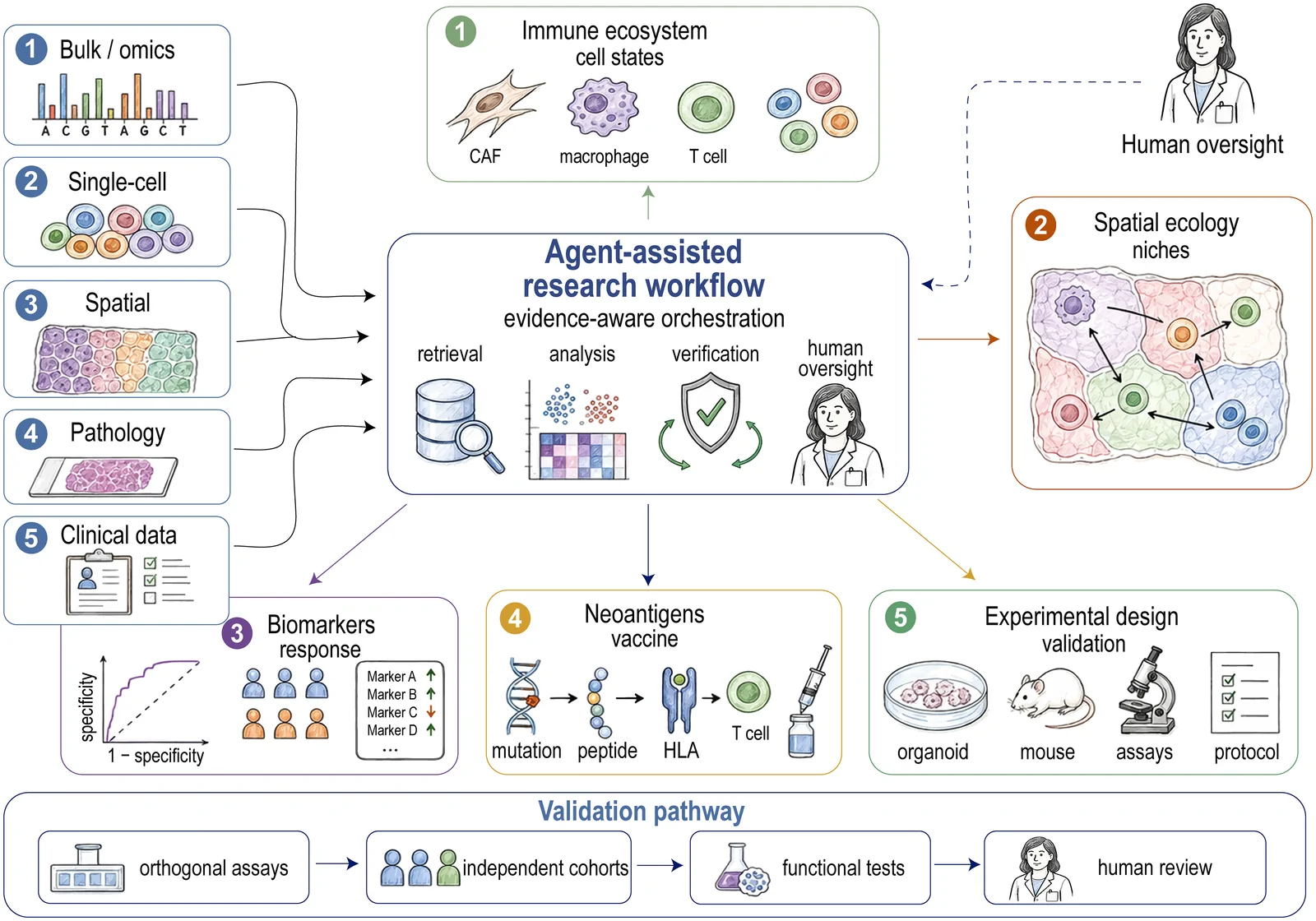
Potential application domains and validation pathways for AI research agents in PDAC tumor immunology. Multimodal molecular, cellular, spatial, pathological, clinical, and experimental data may be coordinated through agent-assisted workflows across five major applications: immune-ecosystem reconstruction, spatial immune ecology, biomarker and treatment-response research, neoantigen and personalized-vaccine prioritization, and experimental design. Agentic systems may assist with data integration, analysis, evidence retrieval, and candidate prioritization, while biological interpretation remains dependent on appropriate validation. Independent cohorts, orthogonal tissue measurements, functional immune assays, co-culture or *in vivo* models, and expert review provide complementary validation routes.

### 4.1 Agent-assisted reconstruction of the PDAC immune ecosystem

Human PDAC contains heterogeneous stromal, myeloid, and adaptive immune states, including localized immune niches that are not captured by a single tumor-wide label (2, 3, 18). Bulk profiling, single-cell assays, spatial measurements, and pathology therefore provide complementary rather than interchangeable evidence. An agent can coordinate dataset selection, metadata harmonization, quality control, annotation, patient-level subgroup comparisons, and cross-platform checks while preserving tissue site, treatment exposure, assay, and sample provenance. It can also record when a cell-state label changes across annotation references or when a cohort comparison is confounded by platform or treatment context. The patient remains the inferential unit: cells, fields, and regions can refine a patient’s representation but should not be counted as independent replicates (43). Cross-modal agreement should be stated at the level actually measured, rather than collapsed into a claim that one modality confirms another. A useful output is not a larger catalogue of cell labels, but a traceable patient-level account of which states recur across modalities, where they occur, and which observations justify a candidate mechanism.

CellAtria supplies direct technical workflow evidence for this application because its evaluation included pancreatic-cancer single-cell datasets and assessed a connected sequence of data discovery, retrieval, standardization, and analysis with provenance capture (9). The evaluated result concerns workflow execution. It does not show that CellAtria identified a new PDAC immune mechanism or validated one experimentally. Within a PDAC study, its role could be tested through patient-level agreement with prespecified annotations, robustness to dataset and reference changes, and concordance with orthogonal protein or tissue measurements. A biologically meaningful test would also examine whether the workflow preserves sample hierarchy and treatment context when integrating studies. Discordant annotations should remain linked to their reference, evidence, and downstream effect rather than being hidden by a consensus label. Those comparisons belong to the evaluation framework in Section 5; here, CellAtria establishes that a provenance-rich agent workflow can be executed on relevant data and provides a concrete starting point for disease-specific evaluation.

### 4.2 Agent-orchestrated spatial immune ecology and cellular interaction analysis

Spatial research asks where molecular and cellular states occur and how their organization changes across regions, treatments, and disease sites. PDAC studies have resolved marked regional heterogeneity, neural and stromal niches, and differences between primary and metastatic lesions (44–46). A research agent could coordinate image registration, segmentation, cell annotation, neighborhood definition, multimodal alignment, and interaction analysis while retaining the parameters and tissue hierarchy of each step. It could select an analysis appropriate to the platform, detect failed registration or segmentation, compare neighborhood definitions, and return uncertain regions for pathology review. This coordination is valuable because an error in registration or annotation can propagate into a biological interpretation. Regional sampling and spatial autocorrelation must also remain visible when statistics are calculated. Agent-maintained provenance can connect each proposed neighborhood to the image, segmentation, marker set, patient, region-selection rule, and analysis version from which it arose.

SP-Mind was evaluated on spatial proteomics tasks that included PDAC imaging mass-cytometry data, providing direct technical task evidence on PDAC data (40, 47). Its task performance does not demonstrate that an inferred neighborhood has biological function or clinical relevance. Spatial proximity, colocalization, and ligand-receptor inference can nominate a relationship, but none is equivalent to functional interaction. The claim should therefore determine the next evidence: blinded pathology or orthogonal imaging can confirm localization, whereas perturbation, blockade, co-culture, or another functional assay is needed to test a proposed mechanism. Patient-level replication across sections or centers addresses generalizability rather than mechanism. This boundary preserves the value of spatial orchestration without overstating what location alone establishes.

### 4.3 Agent-assisted biomarker discovery and prediction of treatment response

Biomarker research is constrained less by the number of candidate features than by the quality of treated cohorts and outcome labels. A prognostic association describes outcome regardless of treatment; a predictive biomarker requires evidence that treatment benefit differs according to the marker (48). In PDAC, an agent could harmonize treatment exposure, sampling time, response definitions, molecular assays, and clinical covariates; integrate genomic, transcriptomic, immune, and spatial features; and organize evidence supporting each claim. It could identify label conflicts, separate pretreatment from on-treatment samples, and prevent a signature derived from survival in an untreated cohort from being presented as a predictor of immunotherapy benefit. The PRINCE study illustrates the type of treatment and immune context that must remain attached to biomarker interpretation (49). The essential output is a claim-evidence record that distinguishes response prediction from survival association, identifies the population and comparator to which the claim applies, and determines whether the available cohort can answer the proposed question.

Biomni offers adjacent evidence for retrieval, planning, and tool orchestration, not direct evidence of PDAC immunotherapy-response prediction (8). TCGA-PAAD and TIDE can inform hypotheses about molecular context or immune escape, but untreated public cohorts and a general immune-dysfunction framework do not supply authentic PDAC treatment-response labels (33, 50). An agent should preserve that distinction when selecting data and wording conclusions. Where studies use different response criteria, regimens, or sampling schedules, the system should retain the disagreement instead of translating them into one apparently uniform endpoint. Candidate features can then be presented as discovery hypotheses, prognostic factors, or treatment-interaction candidates according to the data actually available. Detailed requirements for patient-level separation, external testing, calibration, and comparator design are addressed in Section 5, where predictive performance can be evaluated without allowing a prognostic signal to inherit a treatment-response claim.

### 4.4 Agent-enabled neoantigen discovery and personalized vaccine development

Neoantigen research is well suited to orchestration because candidate generation depends on a linked sequence of tumor-normal sequencing quality control, variant calling, HLA typing, expression assessment, antigen processing and presentation prediction, peptide ranking, and experimental planning (51). NetMHCpan, MHCflurry, pVACtools, and IEDB provide bounded predictions or knowledge resources; they are tools, not autonomous research agents (52–55). An agent could coordinate these components, retain correlated sources of prediction error, and rank candidates by explicit criteria. It should preserve which filters removed each candidate and whether multiple scores depend on overlapping training data or binding assumptions. PDAC studies provide the biological basis for this application: neoantigen quality and immunoediting are associated with long-term disease behavior, and personalized RNA vaccination has elicited neoantigen-specific T-cell responses in selected patients (56–59). These studies establish PDAC-domain relevance, not agent performance.

Validation should follow the specific claim. Computational candidacy identifies peptides worth testing but does not establish natural presentation or T-cell recognition. Mass-spectrometry immunopeptidomics provides direct evidence that a peptide was presented by HLA under the sampled conditions; a negative result may still miss low-abundance presentation (60). Peptide-MHC multimer staining detects and quantifies T cells with cognate-binding receptors but does not by itself establish functional competence. ELISpot, intracellular cytokine staining, or cytokine release after antigen stimulation supports antigen-triggered functional activation, although peptide-pulsed assays do not prove endogenous processing and presentation by tumor cells. Activation or cytotoxicity against target cells that endogenously process and present the antigen provides stronger evidence of tumor-relevant recognition under the tested conditions. *In vivo* tumor control and patient outcomes address higher-order biological or clinical relevance. These assays interrogate distinct, partly orthogonal evidentiary links and are not interchangeable (61–63). An agent should route each candidate to the assay that tests the outstanding link instead of treating several assays as interchangeable proof.

### 4.5 Agent-assisted experimental design and combination strategy prioritization

Research agents could convert a mechanistic question into a draft experimental object by linking the hypothesis to a model, controls, readouts, and an analysis plan. Relevant PDAC applications include multiplex immunofluorescence, flow cytometry, organoid-immune co-culture, humanized orthotopic models, immune-function assays, and combination studies (22, 23, 25, 64, 65). The agent can retrieve technical standards, check compatibility among samples and reagents, compare model limitations, and maintain a traceable rationale for each choice. It can also expose where a proposed experiment cannot distinguish competing mechanisms or where a model lacks the immune, stromal, or HLA context required by the claim. These are useful design outputs before any protocol is executed. The protocol remains a draft research object until experts confirm feasibility, biosafety, ethics, and the biological assumptions that cannot be resolved computationally.

Biomni provides adjacent evidence for multi-step biomedical planning (8). PDAC organoid, humanized-model, clinical, and stromal-targeting studies provide domain evidence about what can be measured or perturbed, but they did not evaluate agentic prioritization (22, 25, 49, 66). Combination ranking should therefore keep mechanistic plausibility, model-specific efficacy, clinical-stage evidence, and toxicity as separate attributes rather than compressing them into one score. A candidate supported in one model should be paired with the model feature that made the result informative and the evidence needed before extrapolation. Section 5.1 evaluates protocols as research products, including their falsifiability, controls, replication, failure criteria, and feasibility; Section 5.2 determines whether adaptive orchestration improves those products over matched alternatives. **Table 2** maps the five application domains to their present evidence status and the validation pathway appropriate to each claim.

**Table 2:** Applications, evidence status, and validation pathways for autonomous AI research agents in PDAC tumor immunology.

Application area	PDAC immunologic focus	Agent-orchestrated role	Evidence status	Required validation	Critical limitations
Immune microenvironment	Myeloid enrichment; T-cell restriction; CAF heterogeneity; interpatient immune phenotypes	Metadata/QC; cell-state annotation; subgroup comparison; infiltration estimation; ligand-receptor prioritization	PDAC-domain evidence (2, 3, 11); direct technical agent evidence on pancreatic-cancer data: CellAtria (9), workflow level; biological/clinical validation not established	Independent cohort; patient-level models; flow cytometry; mIF/IHC	Dissociation/batch bias; marker dependence; pseudoreplication; inferred communication requires functional confirmation
Spatial immune ecology	Immune infiltration/exclusion; stromal retention; TLS; neural invasion; primary/metastatic differences	Registration/segmentation; annotation; neighborhood/ecotype analysis; multimodal integration	PDAC-domain evidence (17, 44–47); direct technical agent evidence on PDAC data: SP-Mind (40), task level; biological/clinical validation not established	Blinded pathology review; independent cohort; mIF/IHC; blockade/co-culture	Spatial autocorrelation; region selection; within-patient pseudoreplication; multiple testing; colocalization does not establish function
Biomarkers and treatment response	Prognostic vs predictive biomarkers; immune-escape/resistance mechanisms	Data/outcome harmonization; model development; mechanism retrieval; run-log management	PDAC treatment evidence (49); adjacent agent evidence: Biomni (8); direct PDAC-agent predictive/clinical validation not established	Patient-level splitting; external validation; ROC/PR-AUC; calibration; treatment interaction	Authentic-label scarcity; leakage; small/class-imbalanced cohorts; overfitting; discrimination does not establish clinical utility
Neoantigens and personalized vaccines	Expression, clonality, antigen processing/presentation, T-cell recognition; vaccine-target selection	Paired-sample QC; variant/HLA analysis; peptide ranking; evidence retrieval; validation planning	PDAC-domain evidence (56–59); prospective application; end-to-end PDAC-agent validation not established	Natural HLA presentation (immunopeptidomics); antigen-specific T-cell detection (pMHC multimer); functional activation (ELISpot/cytokine/ICS); endogenous target-cell recognition/cytotoxicity; *in vivo*/clinical relevance (60, 61)	Training dependence; correlated errors; presentation is not T-cell recognition; multimer binding is not function; peptide-pulsed activation is not endogenous tumor recognition; *in vitro* recognition is not clinical efficacy
Experimental design and combination strategies	Falsifiable protocols; model choice; evidence maturity; toxicity/feasibility	Protocol drafting for mIF/IHC, flow cytometry, co-culture and humanized mice; evidence stratification	PDAC-domain/standards evidence (22, 23, 25, 49, 64–66, 70); adjacent agent evidence: Biomni (8); PDAC protocol validation not established	Expert review; pilot studies; randomization/blinding; sample-size justification; ethics/biosafety review	Fabricated reagents/doses; species/HLA mismatch; cross-model extrapolation; misuse as treatment advice

Evidence categories distinguish PDAC-domain evidence, direct technical agent evidence on pancreatic/PDAC data, adjacent agent evidence, and prospective application. Direct technical performance does not establish biological discovery, experimental validation, or clinical validity. TIDE is not a PDAC treatment-response label; spatial colocalization does not establish functional interaction; and neoantigen assays test distinct, partly orthogonal evidentiary links.

## 5 Evaluation and validation of autonomous AI research agents in PDAC immunology

General biomedical demonstrations do not establish whether an agent can produce reliable, reproducible, and experimentally useful work in PDAC immunology. Disease-specific evaluation must test the tasks that matter, define the evidence required for each output, and separate the contributions of tools, adaptive orchestration, and human intervention. We therefore propose a preliminary PDAC-specific framework built around three task classes, matched component analysis, native real-world effectiveness, reproducibility, expert adjudication, and bounded experimental execution. It is a methodological proposal rather than a standardized or externally validated benchmark. Its central purpose is to make claims attributable: a performance difference should be linked to the evaluated task and configuration instead of being assigned to an undifferentiated “agent.” This design also aligns the evaluation endpoint with the scientific product, whether that product is a numerical result, an evidence synthesis, a protocol, or an experimentally tested claim. The proposed evaluation framework is summarized in **Figure 5**.

**Figure 5:**
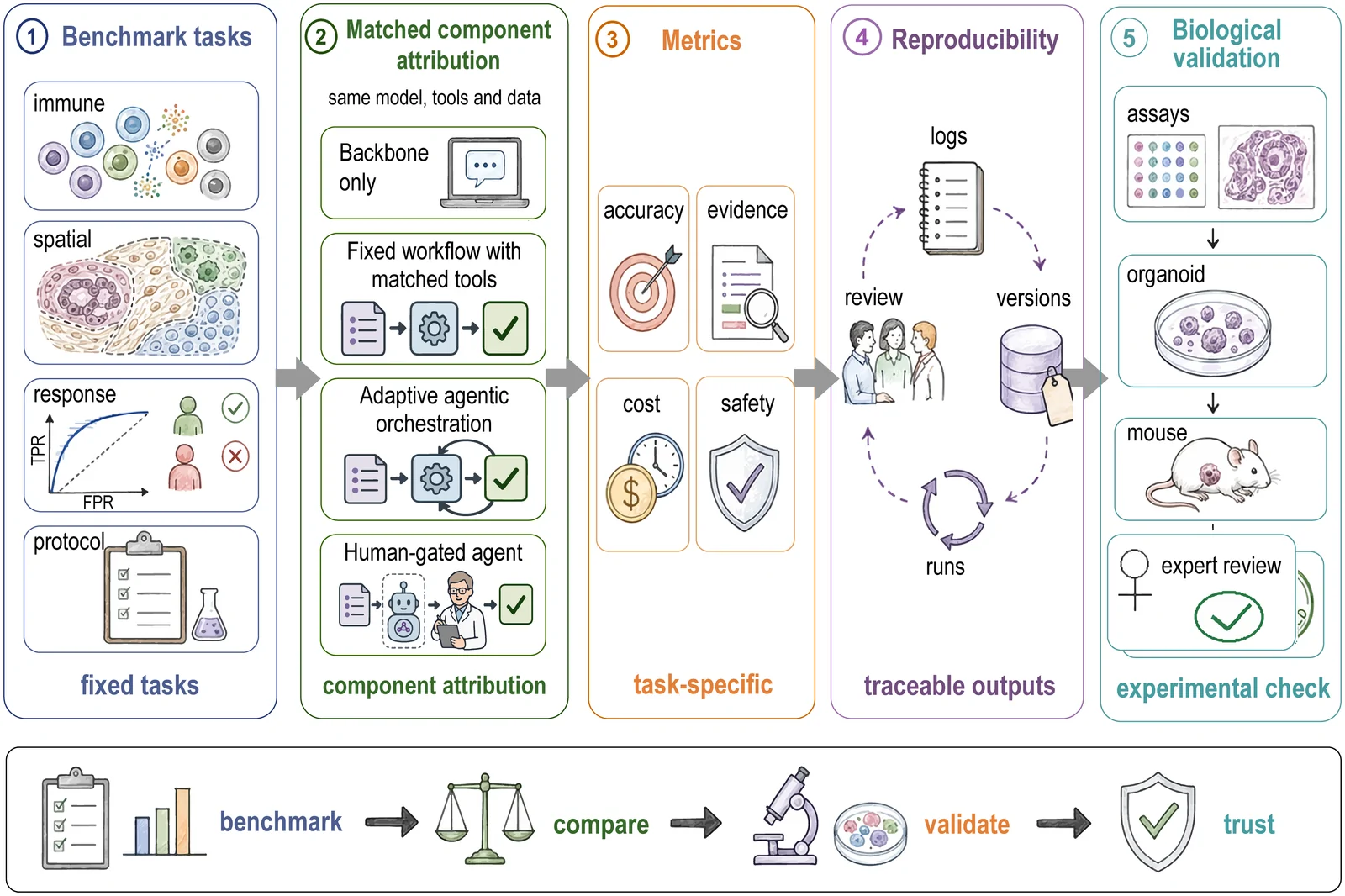
Proposed framework for evaluating autonomous AI research agents in PDAC immunology. The framework integrates disease-relevant tasks, matched component attribution, task-specific metrics, reproducibility assessment, and biological validation. Under matched model, tool, data-access, retrieval, retry, and resource conditions, the key comparison is a fixed tool-enabled workflow versus adaptive agentic orchestration; a separate human-gated condition estimates the effect of a prespecified intervention policy, while a human-only baseline provides contextual performance. Native configurations are compared with compute, cost, latency, tool use, failures, and human burden reported in full. Versioned logs, repeated runs, blinded expert assessment, and bounded experimental execution provide complementary evidence of reproducibility and scientific validity. LLM, large language model.

### 5.1 Benchmark task design

Objective computation, semi-open evidence synthesis, and open-ended protocol design test different failure modes. Objective tasks can include immune-cell deconvolution, single-cell quality control and annotation, or patient-level subgroup analysis with fixed inputs and reference outputs. They reveal whether the system can execute tools correctly, respect the patient hierarchy, and return reproducible objects rather than plausible prose. Semi-open tasks ask the system to assemble and qualify evidence, for example by distinguishing prognostic biomarkers from predictors of treatment benefit or prioritizing combination hypotheses. These tasks should test citation authenticity, claim-evidence fit, recognition of shared study or data lineage, and preservation of unresolved conflict. Open-ended tasks ask for an experimental protocol or panel whose quality cannot be reduced to one reference answer. Together, the classes test computation, evidence judgment, and experimental reasoning without treating them as one capability. Scientific-agent benchmarks provide useful precedents for executable outputs, contamination control, perturbation, and stepwise assessment, although they do not establish PDAC-specific performance (67–69).

Each task should be specified before systems are tested. The specification includes fixed inputs, permitted resources and tools, expected output structure, the reference standard or adjudication rubric, resource limits, and errors that invalidate the result. Objective references may combine simulated truth, orthogonal measurements, and expert-curated labels, with acceptable tolerances defined in advance. Semi-open references should identify the underlying evidence units so that multiple reports of one cohort are not scored as independent corroboration; they should also preserve known disagreements rather than reward synthetic consensus. Reference sets can state which claims are settled, which remain conditional, and which require the system to abstain or request adjudication. Protocol rubrics should assess a falsifiable hypothesis, model suitability, primary readout, positive and negative controls, replication, randomization or blinding where applicable, sample-size rationale, reagent and species or HLA compatibility, statistical analysis, safety, feasibility, stopping or failure criteria, and contingency plans. ARRIVE 2.0 supplies relevant standards for animal work (70). Critical errors, such as fabricated reagents, patient-level leakage, a missing essential control, or treatment advice unsupported by the task, should be reported separately from aggregate quality scores and may trigger task failure regardless of completion.

### 5.2 Comparator design, evaluation metrics, and cost

End-to-end comparisons show whether deployed systems differ, but they do not identify why. If the model, retrieval corpus, tools, number of retries, compute budget, or human correction changes with the system label, the observed difference cannot be assigned specifically to agentic orchestration. This matters because access to a specialized database or an additional expert correction may account for an apparent agent advantage. The framework therefore uses two complementary tracks. A matched component-attribution track estimates incremental contributions under controlled conditions; a native real-world effectiveness track compares systems as deployed. These tracks answer different questions and should not be merged into a single ranking. Both should use frozen tasks and prevent test cases from entering demonstrations, caches, retrieval indexes, or tuning material.

The matched track begins with a common foundation model and a fixed task set. A backbone-only condition has no external tools. A tool-enabled fixed-workflow condition uses the same permitted tools and data access but follows a prespecified sequence. The key contrast adds adaptive planning and replanning while holding the model, tool set, retrieval and data access, resource envelope, retry policy, and stopping rule as constant as feasible. Randomized task order and identical starting context can reduce incidental differences. This contrast estimates the contribution of adaptive orchestration beyond tool availability; if some components cannot be matched, the remaining imbalance must be reported as part of the interpretation. A human-gated condition then adds a prespecified intervention policy to the same agent, defining when experts may approve, correct, redirect, or stop an action. Interventions initiated by the system, required by protocol, and volunteered by reviewers should be distinguished. Comparison with the ungated agent estimates the effect of that specified policy, not a universal benefit of humans. A human-only baseline remains useful as a contextual performance benchmark, but it is not interchangeable with human intervention inside an agent workflow. Component ablations reported for scientific agents and same-backbone tool comparisons provide methodological precedents (41, 67, 71).

The native effectiveness track retains each system’s actual model, tools, memory, retrieval, and interface so that evaluation reflects practical deployment. Results should report task performance alongside compute or token use, elapsed time, tool calls, retries, failure modes, and total cost. Failure reporting should identify where the workflow stopped, whether the system recognized the failure, and whether recovery changed the scientific claim. Human burden requires equal specificity: the frequency, type, duration, timing, and criticality of interventions should be recorded, including whether an intervention corrected a consequential error or completed routine setup. The time required to inspect logs and verify evidence also belongs to the deployed workload. Reporting the full configuration allows readers to judge whether a higher-performing system achieved that result through better orchestration, broader resources, or more expert labor. Native comparisons establish effectiveness of configurations, not causal component attribution.

Metrics should follow the task. Objective computations can use completion, patient-level error, agreement with orthogonal measurements, and between-run variation. Evidence synthesis can use citation authenticity and precision, coverage of prespecified evidence units, claim-strength alignment, dependency recognition, conflict preservation, and critical-error rate. Protocols require blinded expert ratings of falsifiability, controls, feasibility, safety, and statistical coherence. Predictive tasks additionally require discrimination, precision-recall performance for imbalanced outcomes, calibration, confidence intervals, external testing, and a sample size appropriate to the intended model (72–75). Cost and speed should be presented beside scientific quality rather than combined with it, because an inexpensive incorrect synthesis is not efficient research. Paired analyses within tasks and models that account for task and run variability are more informative than an unstructured global score. PROBAST+AI and FUTURE-AI can inform risk-of-bias, applicability, and deployment-oriented reporting without serving as evidence of biological or clinical efficacy (76, 77).

### 5.3 Reproducibility, expert review, and immunologic experimental validation

Reproducibility assessment should treat the execution trace as evidence. Each run should preserve model, tool, database, and code versions; data checksums; prompts or plans; parameters; intermediate outputs; tool errors; retries; evidence dependencies; human interventions; and final claims. The trace should make it possible to distinguish stochastic variation from a change in a resource or decision path. Nondeterministic systems should be run a prespecified number of times, with success rates and output distributions reported rather than selecting the best run. Blinded, independently assigned experts can adjudicate semi-open and open-ended outputs using a frozen rubric. Intraclass correlation is appropriate for continuous ratings, whereas categorical or ordinal outcomes require a suitable kappa statistic (78, 79). Disagreement among experts should be retained and resolved under a prespecified process, because expert consensus is itself an evaluated measurement rather than an automatic ground truth.

Experimental execution can test whether a selected agent-generated protocol is workable and whether its stated biological claim survives the planned assay. Examples include a flow-cytometry panel, multiplex-imaging experiment, organoid-immune co-culture, humanized model, or immune-function assay (22, 23, 25, 64, 65). Researchers perform the wet-lab work and retain responsibility for controls, replication, safety, ethics, and interpretation. A successful experiment supports the executability of that protocol and the biological finding tested under the specified system version, inputs, and conditions. It does not validate every capability of the agent or establish clinical efficacy. Co-Scientist and Robin show how computational hypotheses or returned experimental data can enter broader research loops in adjacent settings (38, 41). **Table 3** translates these principles into proposed tasks, reference standards, metrics, and critical failures for PDAC immunology.

**Table 3:** Proposed evaluation tasks, reference standards, and principal metrics for autonomous AI research agents in PDAC immunology.

Task type	PDAC immunology example	Reference standard	Expected output	Principal metrics	Critical failure risks
Objective computation	Estimate immune-cell composition from bulk transcriptomic data	Simulated/public benchmarks; orthogonal measurements	Proportion matrix; execution log	Completion; correlation/agreement; MAE/RMSE; patient-level error	Reference-matrix mismatch; leakage; estimates treated as ground truth
Objective computation	scRNA-seq QC, annotation, and patient-level subgroup comparison	Expert annotations; prespecified QC; hidden labels	Annotated object; statistics; code	Executability; macro-F1; patient-level effect error	Batch effects; cell-level pseudoreplication; incorrect marker genes
Semi-open evidence synthesis	Prioritize candidate PDAC immunotherapy combinations	Original trials/preclinical studies mapped to underlying evidence units; shared lineages and known discordances	Stratified evidence table; dependency map; unresolved-conflict record; citation trail	Reference authenticity; citation precision/recall; dependency recognition; conflict preservation; claim-strength alignment; critical-error rate	Duplicate reports counted as replication; conflict erased by source counting; cross-cancer/preclinical evidence presented as patient treatment advice
Semi-open evidence synthesis	Distinguish prognostic biomarkers from predictors of immunotherapy benefit	Studies with authentic treatment labels and appropriate comparators; methodological guidance; prespecified conditional/conflicting claims	Claim-evidence matrix with population, treatment, endpoint, evidence dependency, and residual conflict	Classification accuracy; authentic-label use; qualifier preservation; dependency/conflict handling; abstention appropriateness	Survival association, TCGA, or TIDE substituted for response validation; regimen/endpoint differences collapsed; unsupported predictive language
Open-ended protocol	Design a PDAC organoid-immune-cell co-culture experiment	Prespecified expert rubric; technical standards; feasibility/biosafety review	Experimental protocol	Falsifiability; controls; replication; statistics; safety; failure criteria; feasibility	Cell-source/HLA mismatch; fabricated doses; missing controls or failure criteria; cross-model overclaim
Open-ended protocol	Design an mIF/IHC or flow-cytometry immune-phenotyping panel	Formal guidelines; reagent documentation; blinded expert review	Panel; gating/interpretation logic; validation plan	Completeness; spectral/compensation feasibility; control quality; expert agreement	Invalid antibody clones; marker conflicts; missing single-stain/negative controls

This proposed framework is not an established benchmark. Reference standards, primary metrics, and critical errors should be prespecified. Matched component attribution and native real-world effectiveness should be reported separately. Semi-open and open-ended outputs are not patient-level treatment recommendations. MAE, mean absolute error; RMSE, root-mean-square error; F1, F1 score.

## 6 Challenges, open questions, and a roadmap for autonomous AI research agents in PDAC immunology

### 6.1 Current challenges

Long research trajectories magnify local errors. A false factual premise, an unsuitable dataset, an incorrect parameter, or a missed evidence dependency can alter later retrieval, analysis, and prioritization even when each downstream step appears coherent. The system may also produce a well-cited conclusion whose sources share the same underlying cohort or analytical assumption. Biomedical contradiction detection remains an explicit reliability problem (36), and hallucination-detection benchmarks address only part of it (80). System-level reliability therefore depends on checkpoints that examine intermediate claims and actions, calibrated abstention when the evidence or permissions are insufficient, and routes for human takeover. Checkpoints are most useful at irreversible or high-consequence transitions, such as cohort exclusion, external data transfer, target prioritization, or experimental handoff. These mechanisms support bounded execution; they do not convert an uncertain input into reliable evidence.

PDAC also lacks the harmonized data needed for many proposed tasks. Studies differ in disease stage, treatment, sampling time, assay platform, response definition, and endpoint (49, 58), while spatial and single-cell datasets add batch, annotation, and tissue-selection effects (81–83). Data availability is often coupled to clinical and institutional context, making missingness informative rather than random. HLA diversity and site-specific sampling further limit transfer across populations. Sensitive genomic and clinical data create re-identification risks (84), and external tool calls raise questions about data transfer, retention, and auditability. Institutions must therefore define permissible data flows, least-privilege access, responsibility for agent actions, human takeover, biosafety review, and accountability for scientific or clinical interpretation. Permission boundaries must remain effective when tools call other services or write persistent memory. Audit logs also need retention rules and designated reviewers, because collecting a trace does not ensure that consequential actions are examined. In the near term, harmonized disease-specific datasets, metadata, and labels may reduce uncertainty more than additional model scale. The principal challenge categories and corresponding mitigation strategies are summarized in **Table 4**.

**Table 4:** Challenges and actionable mitigation strategies for autonomous AI research agents in PDAC immunology.

Challenge level	Specific problems	Actionable mitigation strategies
Model	Factual hallucinations; immunologic concept errors; tool/parameter errors; long-task instability	Trusted-source/tool whitelists; staged checkpoints; abstention/fallback; evidence trails; immunology-expert review (67–69, 71, 77, 80)
Data	Inconsistent treatment labels; batch effects; platform heterogeneity; within-patient pseudoreplication	Common data model; minimum metadata; patient-level splitting; external-center validation; federated/distributed collaboration with leakage and attack testing (43, 49, 58, 81–83, 85)
Validation	Difficulty advancing computational candidates across *in vitro*, animal, and clinical evidence levels	Prespecified thresholds; orthogonal experiments; failure criteria; preregistration; independent cohorts; prospective validation (25, 64, 65, 70, 76, 89)
Ethics and governance	Re-identification or leakage of genetic/clinical data; excessive permissions; unclear accountability	Data minimization; de-identification/encryption; role-based access; audit logs; human takeover; ethics and biosafety approval (77, 84, 85)

These measures are risk controls or modes of collaboration; they do not guarantee factual correctness, experimental feasibility, or privacy. Multi-agent collaboration, closed-loop experimentation, federated learning, and immune digital twins remain prospective directions requiring validation.

### 6.2 Unresolved questions

A key unmet methodological need is a frozen PDAC task set that combines authentic treatment and outcome labels, cross-platform immune measurements, and an external test cohort for agent evaluation. Deconvolution, single-cell integration, and spatial mapping can vary by method and dataset (81–83), while cell- or field-level analyses can exaggerate precision if the patient hierarchy is ignored (43). A useful reference dataset would link raw and processed data, tissue region, treatment context, patient-level outcomes, and orthogonal measurements while reserving an external set from all system development. It would also document where an apparent ground truth depends on expert annotation or assay sensitivity. Priority should therefore go to patient-level cross-platform datasets with orthogonal tissue or immune references, documented missingness, and prespecified endpoints. Such datasets would resolve more uncertainty than increasing task count without improving the reference standard.

A second unresolved question is how much revalidation should follow a change in the foundation model, prompt policy, tool, database release, retrieval index, workflow code, or permission boundary. Even a database update may change candidate rankings or remove evidence previously used in a conclusion. Update impact should be assessed against the original intended use, critical failure modes, and modified components, with partial and complete revalidation triggers defined before deployment. Previously failed cases, dependency-sensitive syntheses, and high-consequence actions should form a persistent regression set. Monitoring should also detect changes in data availability or tool behavior that occur without a nominal system release. Provenance records must therefore capture versions and configuration changes at a level that supports comparison across releases. PROBAST+AI and FUTURE-AI offer relevant principles for applicability, risk, and lifecycle governance, but they do not supply a validated PDAC-agent revalidation standard (76, 77).

### 6.3 Future directions

The near-term priority is an evidence-aware PDAC immunology agent with a defined intended use, a disease ontology, a curated corpus linked to primary evidence units, and a registry that records provenance, dependency, conflict, and validation status. Initial use cases should be bounded, such as patient-level evidence synthesis or a versioned single-cell workflow, so that references and critical errors can be prespecified. Permissions should be narrow enough to support reliable action, with abstention and human takeover when a request exceeds the available evidence or authorized tools. Frozen tasks and machine-readable logs would make progress measurable without treating maximal autonomy as the objective.

Mid-term development should focus on patient-level alignment of single-cell, spatial, pathology, and clinical data across centers. Common data models, shared endpoint definitions, and explicit mappings among samples, regions, and patients would support external testing, while federated or distributed analysis could enable collaboration when raw data cannot be centralized (85). These strategies must still address site heterogeneity, leakage, privacy attacks, unequal representation, and failures that arise from local platform differences. The research question is whether multimodal orchestration improves a prespecified PDAC inference under external evaluation, not whether more modalities can be added to the workflow.

Long-term systems may connect computational prioritization to human-gated experimental loops. An agent could advance a falsifiable hypothesis to a preregistered experiment, receive structured results, revise the claim, and propose the next bounded action, with stopping rules and independent replication governing progression. The loop should preserve negative and inconclusive results, because selective return of successful experiments would distort subsequent planning. Co-Scientist and Robin provide adjacent precedents for hypothesis development and returned-data iteration (38, 41); they do not validate a closed-loop PDAC immunology system. Immune digital twins may eventually integrate longitudinal tumor, immune, spatial, and pharmacologic processes (86, 87). Such models would require repeated patient-level calibration and a clear distinction between state estimation, outcome prediction, and treatment-effect estimation. The PDAC Molecular Twin is an outcome-prediction platform based on multi-omic data and should not be treated as an immune digital twin or as evidence of treatment effects (88). These longer-term directions remain credible only when each computational and experimental transition carries an explicit evidentiary claim and permission boundary.

The major challenges, unresolved questions, enabling foundations, and future directions for PDAC immunology-specific AI research agents are synthesized in **Figure 6**.

**Figure 6:**
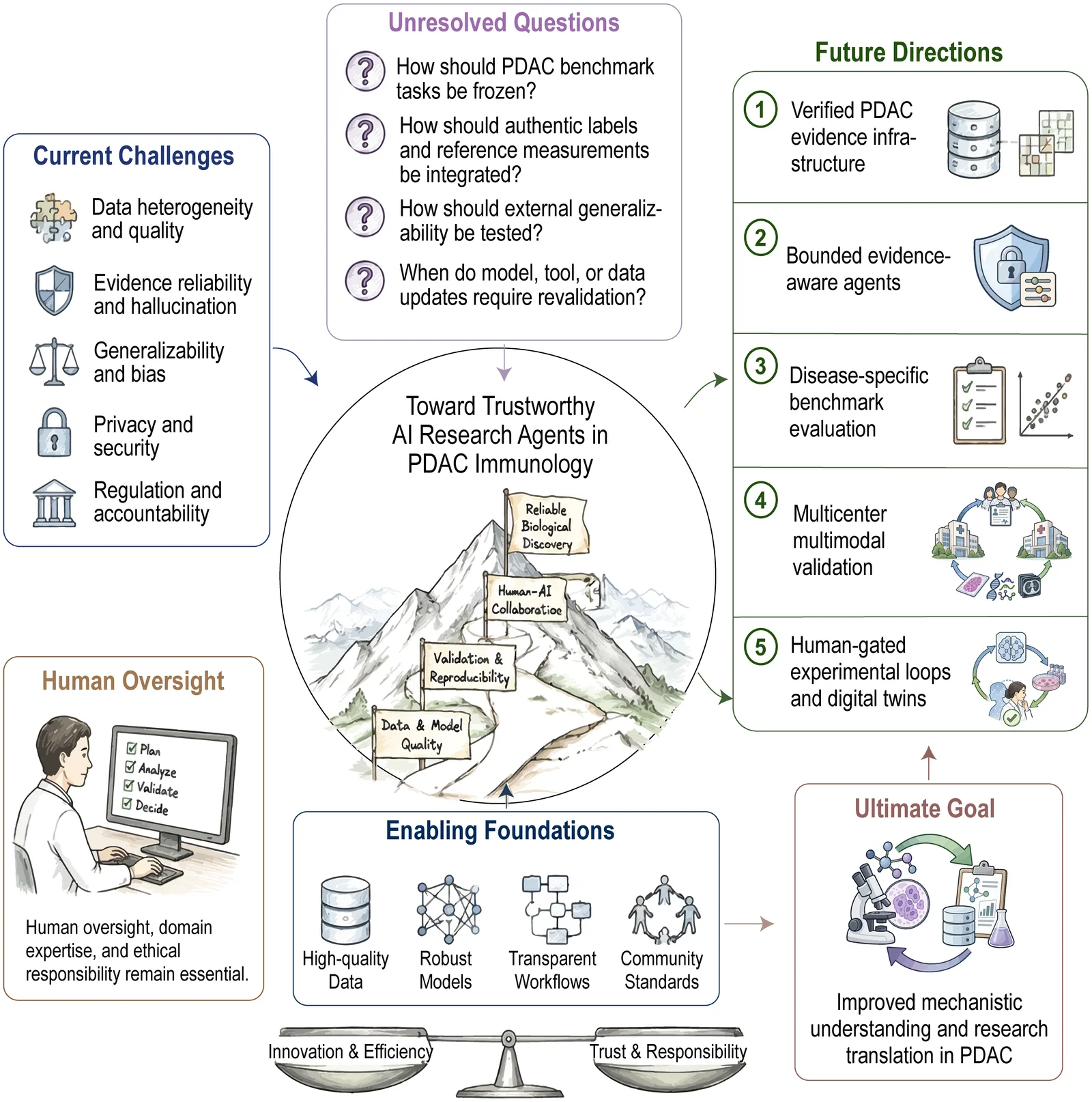
Challenges and roadmap for trustworthy AI research agents in PDAC immunology. Current challenges include data heterogeneity and quality, evidence reliability and hallucination, generalizability and bias, privacy and security, and regulation and accountability. Key unresolved questions concern how PDAC benchmark tasks should be frozen, how authentic labels and reference measurements should be integrated, how external generalizability should be tested, and when model, tool, or data updates require revalidation. Progress will require high-quality data, robust models, transparent workflows, community standards, verified PDAC evidence infrastructure, bounded evidence-aware agents, disease-specific benchmark evaluation, and multicenter multimodal validation. Longer-term directions include human-gated experimental loops and digital twins, with human oversight remaining essential for responsible research translation.

## 7 Conclusion

The principal opportunity for autonomous AI research agents in PDAC immunology is to connect heterogeneous evidence, specialized tools, and experimental routing in an auditable research process. Direct technical studies already show that agent workflows can operate on pancreatic-cancer single-cell data and that agentic spatial tasks can be performed on PDAC data, while adjacent systems demonstrate adaptive planning, verification, and iteration with returned experimental results. The proposed PDAC-specific benchmark turns these capabilities into testable claims by separating task performance, tool access, adaptive orchestration, and prespecified human intervention. It also makes failure scientifically informative by locating whether a claim broke during evidence selection, computation, interpretation, or experimental handoff. Progress should be judged by reliable patient-level inference, transparent dependency and conflict handling, reproducible execution, and experimental work that tests the claim actually made. Under defined human control, constrained autonomy is a scientific strength: it allows adaptive research action without erasing the evidentiary boundaries on which credible PDAC discovery depends. Success is therefore defined by trustworthy research progression, not by the number of decisions a system makes alone.
